# Ubrogepant for the treatment of migraine prodromal symptoms: an exploratory analysis from the randomized phase 3 PRODROME trial

**DOI:** 10.1038/s41591-025-03679-7

**Published:** 2025-05-12

**Authors:** Peter J. Goadsby, Jessica Ailani, David W. Dodick, Amaal J. Starling, Chengcheng Liu, Yingyi Liu, Sung Yun Yu, Jonathan H. Smith, Elimor Brand-Schieber, Joel M. Trugman

**Affiliations:** 1 National Institute for Health Research King’s Clinical Research Facility, King’s College London, London, UK; 2 Department of Neurology, University of California, Los Angeles, CA USA; 3 MedStar Georgetown University Hospital Headache Center, Department of Neurology, Washington, DC USA; 4 Mayo Clinic, Scottsdale, AZ USA; 5 AbbVie, North Chicago, IL USA

**Keywords:** Medical research, Migraine

## Abstract

PRODROME was a phase 3, placebo-controlled, double-blind crossover trial evaluating whether ubrogepant 100 mg, a calcitonin gene-related peptide receptor antagonist, dosed during the premonitory (prodromal) phase of migraine, prevented development of headache and resolved prodromal symptoms. Qualifying prodromal events were defined as attacks with symptoms in which the participant was confident headache would follow within 1–6 h. Of 1,087 screened participants, 477 formed the efficacy analysis population. Outcomes were collected across 48 h showing, for example, at 2 h post-dose, absence of photophobia in 19.5% and 12.5% of ubrogepant- and placebo-treated events, respectively (odds ratio (OR) = 1.72 (95% confidence interval (CI) = 1.13–2.61)); at 3 h post-dose, absence of fatigue occurred in 27.3% and 16.8% (OR = 1.85 (95% CI = 1.17–2.92)) and absence of neck pain in 28.9% and 15.9% (OR = 2.04 (95% CI = 1.25–3.32)) of events; at 4 h post-dose, absence of phonophobia in 50.7% and 35.8% (OR = 1.97 (95% CI = 1.38–2.80)) of events; and at 24 h post-dose, absence of dizziness in 88.5% and 82.3% (OR = 1.82 (95% CI = 1.00–3.30)) of events. At 1 h and 6 h post-dose, respectively, absence of difficulty concentrating occurred in 8.7% and 2.1% (OR = 4.26 (95% CI = 1.17–15.54)) and absence of difficulty thinking occurred in 56.9% and 41.8% (OR = 2.05 (95% CI = 1.14–3.71)) of events. Treatment with ubrogepant during the prodromal phase may ameliorate common prodromal symptoms, with improvements possibly as early as 1 h post-dose.

## Abstract (web summary)

Analysis of the phase 3 PRODROME trial reveals that treatment with ubrogepant during the prodromal phase of migraine may ameliorate common prodromal symptoms, with improvements as early as 1 hour after dose administration.

## Main

Migraine is a common, globally recognized^1^, neurological disorder characterized by recurrent disabling attacks involving headache and symptoms of brain dysfunction^2^. The attack has recognized phases: premonitory (prodrome), aura, headache and postdrome, which, although distinctive, can overlap^3^. The canonical manifestations of an attack—lateralized, throbbing headache with associated sensitivity to light (photophobia), sound (phonophobia) and head movement^4^—have received considerable attention for the last three decades as treatments aimed at the headache phase of the attack were developed^5^. The identification of the role of calcitonin gene-related peptide (CGRP)^6,7^ and the utility of CGRP blockers for both acute and preventive treatment of migraine^5^ offer the possibility of exploring migraine pathophysiology with new tools.

Migraine symptoms that can occur in the premonitory phase (prodrome) fall broadly into three groups: first, higher center, such as cognitive impairment, manifests as difficulty concentrating or thinking—brain fog as it is often labeled by patients—and fatigue. Second, symptoms broadly reflect homeostatic dysfunction, such as food cravings or polyuria and, third, symptoms usually associated with the headache phase can occur in the premonitory phase, such as sensitivity to light (photophobia) or sound (phonophobia)^8^. Prodromal symptoms are highly predictive of impending headache^9^ and are generally considered to be common when enquired after in adults^10,11^ and children^12,13^. Functional brain imaging has identified activations in the central nervous system, such as the hypothalamic region^14,15^, in the premonitory phase. Mapping symptoms to imaging findings suggests central nervous system origins for the migraine attack^16^, with implications for understanding the pathophysiology and, importantly, where to target therapies.

Therapeutics in migraine have advanced considerably over the last three decades^3^. It has been argued whether the origin of both the attack and the pain is peripheral or central^17^, and thus how best to target therapies. Moreover, previous studies of therapeutics in the acute treatment of the premonitory phase (prodrome) have focused on the potential for blocking the onset of headache^18,19^. The analysis of the prevention of headache in the PRODROME study has demonstrated that ubrogepant given in the prodromal phase prevented moderate or severe headache, when compared with placebo, for the following 48 h (ref. ^20^). In the present study, we report the effect of ubrogepant, a small-molecule CGRP receptor antagonist^21^, on the prodromal symptoms commonly reported by the study participants, a prespecified additional endpoint of the PRODROME trial. In addition to the clinical benefit of resolving these symptoms, the findings may offer insights into the role of the central nervous system in the treatment of migraine. The work has been presented in preliminary form at the 75th Annual Meeting of the American Academy of Neurology (Boston, MA, 22–27 April 2023)^22^.

## Results

### Participants and baseline characteristics

**Fig. 1:**
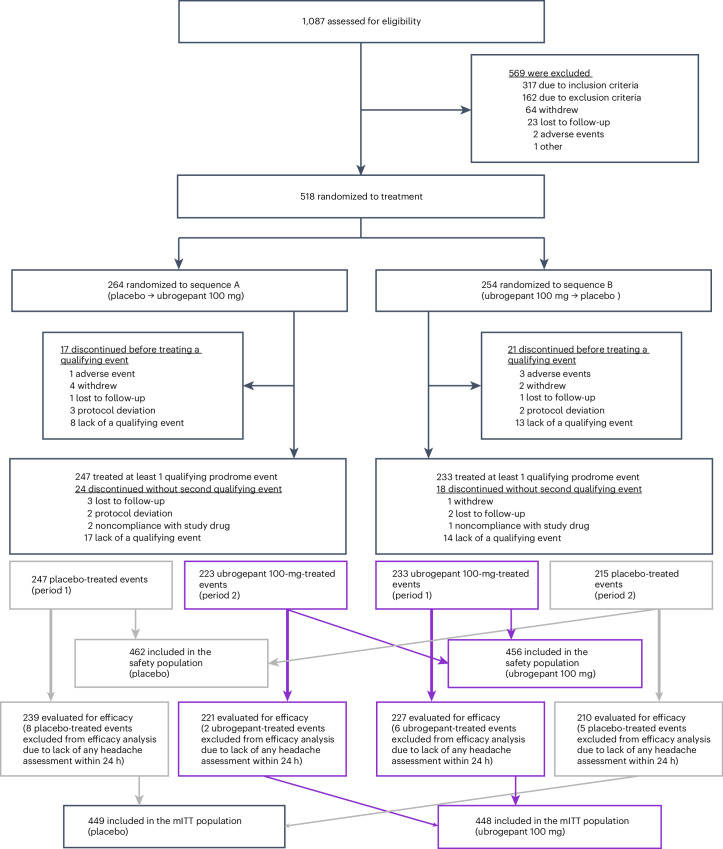
Participant flow. Purple boxes and arrows indicate the number and flow of qualifying prodrome events treated with ubrogepant. Gray boxes and arrows indicate the number and flow of qualifying prodrome events treated with placebo. mITT, modified intention-to-treat.

A total of 1,087 participants were screened, with 518 randomly assigned to double-blind crossover treatment (Fig. 1). The safety and modified intention-to-treat (mITT) populations included 480 and 477 participants, respectively. Participants had a mean age of 42.3 years (Table 1). Most of the participants in the safety population were female (87.7%, *n* = 421 of 480), with most identifying as White (88.1%, *n* = 423 or 480) and non-Hispanic (92.7%, *n* = 445 or 480; Table 1). Of the 518 randomized participants, 84.6% (*n* = 438) completed the trial and 15.4% (*n* = 80) discontinued. Lack of treating two ‘qualifying prodrome events’ within the 60-d double-blind treatment period was the most common reason for discontinuation (10.0%, *n* = 52 of 518). Additional information about PRODROME participants has been previously reported^20^.

**Table 1:** Baseline demographics and migraine characteristics by treatment sequence (safety population)

Demographic or characteristic	Sequence A: placebo or ubrogepant 100 mg (*n* = 247)	Sequence B: ubrogepant 100 mg or placebo (*n* = 233)	Total (*n* = 480)
Age, mean (s.d.), years	41.7 (12.6)	42.9 (13.1)	42.3 (12.9)
Sex, *n* (%)			
Male	31 (12.6)	28 (12.0)	59 (12.3)
Female	216 (87.4)	205 (88.0)	421 (87.7)
Race, *n* (%)			
White	214 (86.6)	209 (89.7)	423 (88.1)
Black or African American	22 (8.9)	15 (6.4)	37 (7.7)
Asian	7 (2.8)	4 (1.7)	11 (2.3)
Native Hawaiian or other Pacific Islander	1 (0.4)	1 (0.4)	2 (0.4)
Multiple^a^	2 (0.8)	4 (1.7)	6 (1.3)
Missing	1 (0.4)	0	1 (0.2)
Ethnicity, *n* (%)			
Hispanic	17 (6.9)	15 (6.4)	32 (6.7)
Non-Hispanic	229 (92.7)	216 (92.7)	445 (92.7)
Missing	1 (0.4)	2 (0.9)	3 (0.6)
BMI, mean (s.d.), kg per m^2^	28.6 (5.6)	28.1 (5.6)	28.3 (5.6)
Migraine diagnosis, *n* (%)			
With aura	53 (21.5)	42 (18.0)	95 (19.8)
Without aura	105 (42.5)	110 (47.2)	215 (44.8)
Both with and without aura	89 (36.0)	81 (34.8)	170 (35.4)
Duration with migraine, mean (s.d.), years	21.6 (12.6)	23.8 (13.3)	22.7 (13.0)

BMI, body mass index.

^a^Participants who reported multiple races are included only in the ‘multiple’ category.

### Outcomes

The five most common prodromal symptoms identified at baseline (pre-dose) in the double-blind treatment period for placebo-treated (*n* = 449) and ubrogepant 100 mg-treated (*n* = 448) events were photophobia (60.8% and 60.9% of events, respectively), fatigue (50.3% and 50.7%), neck pain or stiffness (40.1% and 40.2%), phonophobia (36.1% and 35.9%) and dizziness (31.0% and 29.0%; Table 2). Although the cognitive impairment symptoms of difficulty concentrating (22.5% and 23.0%) and difficulty thinking (16.5% and 15.4%) were the eighth and eleventh most common prodromal symptoms at baseline before treatment, when considering them as one category ‘either difficulty concentrating or difficulty thinking or both’ was reported in 33.0% of placebo-treated and 32.1% of ubrogepant-treated events, making it the fifth most common prodromal symptom (Table 2). Prodromal symptoms of moderate-to-severe intensity were reported at baseline in 31.7–57.2% of placebo-treated and ubrogepant-treated events: specifically, photophobia (42.9% and 42.1% of events, respectively), fatigue (52.2% and 51.5%), neck pain or stiffness (57.2% and 55.6%), phonophobia (38.9% and 36.0%), dizziness (31.7% and 30.8%), difficulty concentrating (45.5% and 39.8%) and difficulty thinking (45.9% and 42.0%) (Table 2 and Supplementary Table 1).

**Table 2:** Most common prodromal symptoms identified at baseline (pre-dose) by occurrence (mITT population)

Prodromal symptom^a^	Placebo (*n* = 449)	Ubrogepant 100 mg (*n* = 448)
Sensitivity to light, *n* (%)	273 (60.8)	273 (60.9)
Mild	156 (57.1)	158 (57.9)
Moderate	96 (35.2)	98 (35.9)
Severe	21 (7.7)	17 (6.2)
Tired, sleepy or fatigue, *n* (%)	226 (50.3)	227 (50.7)
Mild	108 (47.8)	110 (48.5)
Moderate	97 (42.9)	102 (44.9)
Severe	21 (9.3)	15 (6.6)
Neck pain or stiff neck, *n* (%)	180 (40.1)	180 (40.2)
Mild	77 (42.8)	80 (44.4)
Moderate	88 (48.9)	87 (48.2)
Severe	15 (8.3)	13 (7.2)
Sensitivity to sound, *n* (%)	162 (36.1)	161 (35.9)
Mild	99 (61.1)	103 (64.0)
Moderate	57 (35.2)	52 (32.3)
Severe	6 (3.7)	6 (3.7)
Cognitive impairment, *n* (%)^b^	148 (33.0)	144 (32.1)
Difficulty concentrating	101 (22.5)	103 (23.0)
Mild	55 (54.5)	62 (60.2)
Moderate	43 (42.6)	36 (35.0)
Severe	3 (3.0)	5 (4.9)
Difficulty thinking	74 (16.5)	69 (15.4)
Mild	40 (54.1)	50 (58.0)
Moderate	33 (44.6)	28 (40.6)
Severe	1 (1.4)	1 (1.4)
Dizziness, lightheaded, vertigo or imbalance, *n* (%)	139 (31.0)	130 (29.0)
Mild	95 (68.3)	90 (69.2)
Moderate	40 (28.8)	36 (27.7)
Severe	4 (2.9)	4 (3.1)

Prodromal symptoms identified at baseline are symptoms experienced by participants at the time of the qualifying prodrome event. Percentages of prodromal symptom incidence are based on overall treated events *n* and percentages for the intensity levels are based on that symptom’s *n*.

^a^The numbers and percentages represent participants with the prodromal symptom listed.

^b^Includes either difficulty concentrating or difficulty thinking or both.

**Fig. 2:**
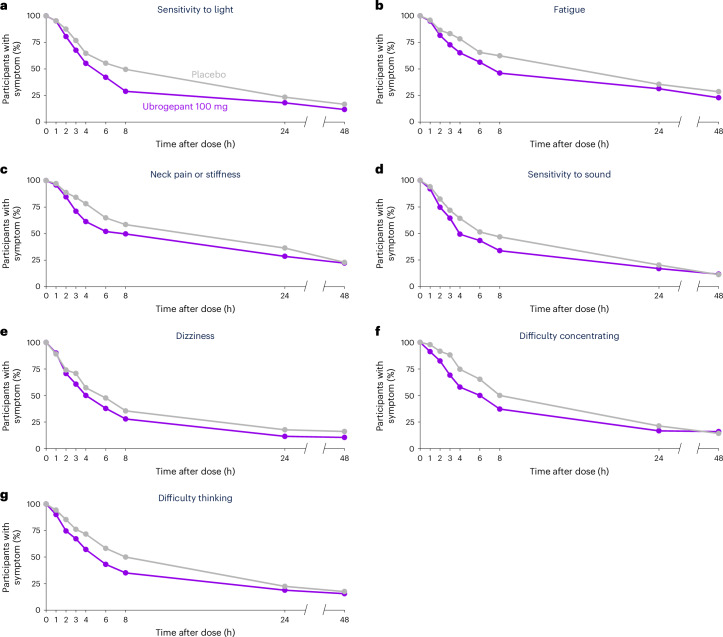
Prodromal symptoms of any intensity for the most common prodromal symptoms at timepoints post-dose. The percentage of participants continuing to have prodromal symptoms at timepoints post-dose are shown. **a**–**g**, Participants who reported the prodromal symptoms at pre-dose: sensitivity to light (placebo, *n* = 273 of 449, ubrogepant 100 mg, *n* = 273 of 448) (**a**); tired, sleepy or fatigue (placebo, *n* = 226 of 449, ubrogepant 100 mg, *n* = 227 of 448) (**b**); neck pain or stiff neck (placebo, *n* = 180 of 449, ubrogepant 100 mg, *n* = 180 of 448) (**c**); sensitivity to sound (placebo, *n* = 162 of 449, ubrogepant 100 mg, *n* = 161 of 448) (**d**); dizziness, lightheaded, vertigo or imbalance (placebo, *n* = 139 of 449, ubrogepant 100 mg, *n* = 130 of 448) (**e**); difficulty concentrating (placebo, *n* = 101 of 449, ubrogepant 100 mg, *n* = 103 of 448) (**f**); and difficulty thinking (placebo, *n* = 74 of 449, ubrogepant 100 mg, *n* = 69 of 448) (**g**).

Among ubrogepant- and placebo-treated events, respectively, 19.5% and 12.5% were associated with an absence of photophobia starting at hour 2 (OR = 1.72 (95% CI = 1.13–2.61)), 27.3% and 16.8% with an absence of fatigue starting at hour 3 (OR = 1.85 (95% CI = 1.17–2.92)), 28.9% and 15.9% with an absence of neck pain starting at hour 3 (OR = 2.04 (95% CI = 1.25–3.32)), 50.7% and 35.8% with an absence of phonophobia starting at hour 4 (OR = 1.97 (95% CI = 1.38–2.80)) and 88.5% and 82.3% with an absence of dizziness at 24 h post-dose (OR = 1.82 (95% CI = 1.00–3.30)) (Fig. 2 and Supplementary Table 2). An absence of cognitive symptoms was also observed: as early as 1 h post-dose for difficulty concentrating in 8.7% and 2.1% of ubrogepant- and placebo-treated events, respectively (OR = 4.26 (95% CI = 1.17–15.54)) and at 6 h for difficulty thinking in 56.9% and 41.8% (OR = 2.05 (95% CI = 1.14–3.71)) of events (Fig. 2 and Supplementary Table 2).

Analysis censoring data collected after rescue medication use for headache (if needed) yielded similar results (Supplementary Table 3 and Supplementary Fig. 2). Rescue medication use within 24 h post-dose was 21.7% for ubrogepant-treated events compared with 39.4% for placebo-treated events (Supplementary Table 4).

### Safety

Treatment-emergent adverse events (TEAEs) were reported for 55 of 462 (12%) qualifying prodrome events treated with placebo and 77 of 456 (17%) events treated with ubrogepant 100 mg within 48 h of administration. Treatment-related TEAEs were identified in 42 of 462 (9%) of placebo-treated events and 60 of 456 (13%) ubrogepant 100 mg-treated events. As previously reported^20^, there were no adverse events (AEs) leading to study discontinuation and no serious AEs. The most common AEs (≥2%) after placebo and ubrogepant administration were nausea (3% versus 5%), fatigue (2% versus 3%), dizziness (3% versus 2%) and somnolence (1% versus 2%).

## Discussion

The results suggest that ubrogepant, a gepant, CGRP receptor antagonist, administered in the premonitory (prodromal) phase of migraine, when headache is absent, may promote the resolution of common prodromal symptoms. Moreover, in this trial, 31.7–57.2% of premonitory symptoms were moderate to severe in intensity and associated with functional disability^20,23^. As premonitory symptoms can be disabling, their treatment alone is clinically relevant, beyond the consideration that treatment during the prodrome prevents headache onset and improves function over 24–48 h, as demonstrated in the primary analysis of the study^20^. Greater awareness of the clinical symptomatology of the prodromal phase, as well as the availability of effective treatment, offers a major opportunity to improve the treatment of acute migraine.

It is interesting to examine the time course and magnitude of efficacy of ubrogepant for the different prodromal symptoms. The most common symptom, photophobia, which was present in approximately 61% of qualifying prodromal events, appeared to resolve after treatment with ubrogepant as early as 2 h post-dose, with a consistent effect across the first 8 h and an effect still evident at 48 h post-dose. Although being reported by only approximately 22% of participants, difficulty concentrating may also benefit from ubrogepant treatment, with resolution as early as 1 h post-dose and up to 6 h, and the trend continuing to 24 h post-dose. In contrast, ubrogepant appeared to have limited impact on the resolution of dizziness, which was present in approximately 30% of qualifying prodromal events, and these effects were observed primarily at the 24-h timepoint. Given the varied pathophysiology of the different symptoms, one would not expect ubrogepant to be equally effective or have a similar time course for all prodromal symptoms. Still, similar trends were observed overall for prodromal symptom resolution, which appeared to occur more frequently with ubrogepant than placebo.

The symptoms of the premonitory (prodromal) phase of a migraine attack have been recognized for more than a century^24^. In a case series of 50 patients, 17 identified symptoms on the day before their headache^25^, whereas a larger series of 530 reported 160 patients identifying premonitory (prodromal) symptoms and speculated on a role of the hypothalamus in the pathophysiology^26^. Studies based on clinic populations in adults^10,11,27^ and children^12,13^ and on large cross-sectional material^28^ have consistently identified premonitory symptoms, such as fatigue and cognitive dysfunction. In patients who recognize these symptoms, they are highly predictive of impending headache^9^, although the reliability of prediction remains an issue^29^.

Many of these common premonitory symptoms, such as photophobia, fatigue, phonophobia, cognitive impairment—difficulty concentrating or difficulty thinking, or both—and dizziness, may well originate in the brain. It has long been argued whether migraine is primarily a disease of the brain or of peripheral, specifically vascular, origin^30^. The new data firmly support a brain origin for migraine attacks. Both symptoms of the premonitory phase, such as photophobia and thirst or food cravings, and outcomes from functional imaging studies implicate brain regions in the earliest phase of a migraine attack^31^. As an example, laboratory studies implicating neuropeptide Y in the modulation of trigeminovascular nociceptive traffic^32^, and its localization in the hypothalamus^33^, or studies of ventral tegmental area reward mechanisms and their interaction with trigeminovascular nociception^34^ offer support to neuroimaging studies that demonstrate activation of these regions in the premonitory phase of an attack^14,15,35^. Photophobia, a symptom classically associated with the headache phase of migraine^4^, may also be reported in the premonitory (prodromal) phase and is indeed the most commonly reported symptom in the present study. Brain imaging has shown a signature in the visual cortex for premonitory phase photophobia^36^, demonstrating a central nervous system-associated biological change. Similarly, brainstem sites, such as the rostral ventromedial medulla, are implicated in trigeminovascular nociceptive modulation^37^ with variation over the cycling of migraine^38^.

There remains considerable debate around the site of action of anti-migraine treatments. The triptans, serotonin 5-HT_1B/1D_ receptor agonists, were developed on the inchoate basis that they constricted dilated cranial blood vessels which were the cause of the pain^39^. Several findings, including dissociation of the timing of a vascular effect and the pain response^40^, and the neural effect of the drugs^41^, have led to the broad adoption of a view that they act primarily through a neural target^42^. Although sumatriptan can be measured in the cerebrospinal fluid^43^, and the exclusively neurally acting ditans, 5-HT_1F_ receptor agonists^44^ are both effective^45^ and have brain access^46^, migraine therapies are widely considered to act outside the brain. A positron emission tomography study of telcagepant showed low receptor occupancy after a less than optimal dose of 140 mg^47,48^, leaving unresolved the issue of whether there was potential for gepants to have effects in the brain. Although it may be argued that the effects of ubrogepant in the present study on photophobia and phonophobia or neck discomfort reflect a primary peripheral site of action with brain consequences, it is difficult to argue that position for cognitive dysfunction, leaving a brain site of action as a more attractive, overall hypothesis. Broadly, the findings of the clinical trial support imaging studies that have identified central nervous system sites as the locus of initiation of a migraine attack.

## Limitations

The participants studied could identify prodromal symptoms that were reliably followed by headache as determined via a rigorous screening procedure^20^. Clinical experience suggests that it is not complex to point out these prodromal symptoms to patients and offer the possibility of the association, which patients can then recognize. Nevertheless, widespread adoption of this approach will require further real-world experience. Whether gepant intervention alters migraine aura or influences the postdrome^49^ was not rigorously explored in the present study and offers interesting avenues for insights and treatment opportunities in the future.

The primary objective of the PRODROME trial was to evaluate the efficacy of ubrogepant on the occurrence of headache after administration during the prodrome phase of a migraine attack. The trial was not primarily designed to evaluate the impact of ubrogepant on resolution of prodromal symptoms; these endpoints were prospectively defined as additional endpoints in the protocol. These analyses were outside the hierarchical gatekeeping procedure to control for type-1 error and were therefore not controlled for multiple comparisons. Although our results suggest a consistent amelioration of common prodromal symptoms after ubrogepant compared with placebo, additional studies specifically designed to evaluate the effect of acute treatment on prodromal symptoms are warranted.

Additional limitations resulting from the study design include participants being instructed to administer the study drug when they felt confident that a headache would follow within 1–6 h, and not at the onset, or first recognition, of prodromal symptoms. Furthermore, we did not analyze differences in symptom resolution between those who did and those who did not develop headache. Analyses censoring data collected after rescue medication use for headache (if needed) showed results similar to the uncensored data, suggesting that a difference in rescue medication use between ubrogepant and placebo treatment groups was probably not a confounding factor (Supplementary Table 3 and Supplementary Fig. 2).

## Conclusion

Based on this double-blind, placebo-controlled trial, ubrogepant 100 mg, when administered in the premonitory (prodromal) phase of migraine, before a headache has commenced, may treat symptoms such as photophobia, phonophobia and cognitive dysfunction. The clinical phenotype of the premonitory phase, its functional neuroimaging findings and the corroborating experimental neurobiology all point to a pathophysiology in the central nervous system. Reversing these symptoms with a gepant emphasizes the importance of the brain in migraine and offers the promethean possibility that targeting central nervous system mechanisms will be a fruitful path to new therapeutics in this common and disabling disorder.

## Methods

### Trial design

The trial design for the PRODROME study has been previously reported^20^. PRODROME (NCT04492020) was a phase 3, multicenter, randomized, double-blind, placebo-controlled, crossover trial conducted from 21 August 2020 through 19 April 2022, at 75 research centers and headache clinics in the United States of America (Supplementary Table 5). After a 60-d screening period, participants with migraine, who could reliably identify migraine attacks with prodromal symptoms that were reliably followed by headache within 1–6 h at least 75% of the time, were randomized to sequence A or B in a 1:1 ratio and entered the double-blind treatment period, during which they were tasked to treat two qualifying prodrome events within a 60-d period (Supplementary Fig. 1). A qualifying prodrome event was defined by the presence of prodromal symptoms that the participant was confident would be followed by a headache within 1–6 h. Headache should not be present at the time of a qualifying prodrome event and the participant should not have had a headache in the previous 48 h. Acute treatment should not have been used within 48 h before the qualifying prodrome event. Defining a qualifying prodrome event as 1–6 h before the expected headache onset was done to provide time for the acute treatment to take effect and aid in differentiating aura symptoms from prodromal symptoms.

Participants in sequence A received placebo to treat the first qualifying prodrome event and ubrogepant 100 mg to treat the second qualifying event, whereas participants in sequence B received ubrogepant 100 mg to treat the first qualifying prodrome event and placebo to treat the second qualifying event. The two treatments had at least 7 d of washout in between.

During the double-blind study period, after taking the study drug, rescue medication was permitted only after headache onset. For a mild headache, rescue medication was permitted 24 h after headache onset. For a headache of moderate or severe intensity, rescue medication was permitted at any time after headache onset. No rescue medication was permitted during the qualifying prodrome event if a headache had not yet occurred. Permitted rescue medications included nonsteroidal anti-inflammatory drugs, acetaminophen, triptans, ditans, ergots, analgesics or combination analgesics, opioids or antiemetics.

During screening, participants identified and confirmed prodromal symptoms from a predetermined list of 30 symptoms. This list included common and uncommon prodromal symptoms, as well as the options ‘other’ and ‘a feeling, not otherwise described or difficult to describe’.

An automated interactive web response system was used to manage randomization, which occurred at visit 2. To maintain the blind, identical blister cards were dispensed at visit 2 and the visit after treatment of the first qualifying prodrome event (visit 3). Each blister card contained two tablets of either placebo or ubrogepant 50 mg. Participants in treatment sequence A received a card with placebo at visit 2 and ubrogepant 100 mg at visit 3, whereas participants in treatment sequence B received a card with ubrogepant 100 mg at visit 2 and placebo at visit 3. During the double-blind treatment period, each participant was instructed to take the study drug for each qualifying prodrome event as soon as they were confident that a headache would inevitably follow within 1–6 h.

### Trial participants

Eligible participants were adults (aged 18–75 years), female or male (based on self-report) with at least a 1-year history of migraine with or without aura consistent with a diagnosis according to the *International Classification of Headache Disorders*, 3rd edn^4^ and a history of two to eight migraine attacks per month with moderate-to-severe headache in each of the 3 months before screening. At the screening visit, participants were asked whether they could identify migraine attacks in which prodromal symptoms were present and likely to be followed by headache within 1–6 h at least 75% of the time. If participants answered in the affirmative, participants received an eDiary to record all their qualifying prodrome events during the 60-d screening period. To be eligible for randomization, participants were required to record 3–16 qualifying prodrome events during the 60-d screening period, with at least 75% followed by a headache, of any intensity, within 1–6 h.

Exclusion criteria have been reported previously^20^. Briefly, exclusion criteria included: history of clinically important hematological, endocrine, pulmonary, renal, hepatic, gastrointestinal or neurological disease that has not been stable for at least 1 year, history of cardiovascular, cerebrovascular or neurological disease, confounding psychiatric conditions including dementia or epilepsy, difficulty distinguishing migraine from other tension type or other headache types, chronic migraine, trigeminal autonomic cephalalgia, painful cranial neuropathy, overuse of medication for migraine (opioids or barbiturates >2 d per month, triptans or ergots ≥10 d per month, simple analgesics ≥15 d per month) within the last 3 months or previous CGRP injectable use within the previous 3 months.

### Outcome measures

The primary endpoint of the PRODROME trial was absence of moderate or severe intensity headache within 24 h post-dose. Prespecified additional endpoints (that is, exploratory endpoints) were the absence of the five most common prodromal symptoms, of any intensity (mild, moderate or severe), at each of the prespecified timepoints (1, 2, 3, 4, 6, 8, 24 and 48 h), regardless of the presence of headache. Additional post-hoc analyses were performed on cognitive impairment symptoms, difficulty thinking or difficulty concentrating, or both. After identifying a qualifying prodrome event in which the participant was confident that a headache would follow within 1–6 h, the participant entered their premonitory (prodrome) symptoms in the eDiary and reported the presence or absence of those prodromal symptoms (up to six patient-identified symptoms were preprogrammed into the eDiary for ease) at the prespecified timepoints. Entries could not be altered retrospectively. After treating the qualifying prodrome event with study medication and completing their initial eDiary entry, participants were required to report the absence or presence of a headache and prodromal symptoms at each timepoint post-dose. Safety assessments included the incidence of adverse events, clinical laboratory tests, electrocardiograms, vital signs, physical examination and the Columbia Suicide Severity Rating Scale. Adverse events were collected from the time of informed consent until 30 d after the last dose. An adverse event was considered to be a TEAE if the adverse event began or worsened (increased in severity or became serious) on or after the date of the first dose of study drug. The safety population included all treated participants who took at least one administration of study drug.

### Statistical analysis

All efficacy analyses used the mITT population, defined as all randomized participants with one or more assessments of headache occurrence within 24 h after taking a double-blind study drug for one or more qualifying prodromal events during the double-blind treatment period. The mITT population is defined in the protocol and is equivalent to a full analysis set consistent with the International Council of Harmonisation (ICH) guidelines^50^. The safety population included all treated participants who took one or more administrations of study drug. Determination of sample size was previously reported; 480 participants in the mITT population were estimated to provide 95% power to determine a 16-point treatment difference in response rate for the primary endpoint, using a two-sided 5% significance level^20^.

For each of the five most common prodromal symptoms, as well as difficulty concentrating and difficulty thinking, the absence of symptoms of any intensity at each timepoint was analyzed using a generalized linear mixed model (GLMM) in the observed binary response variable. ORs (95% CIs) were based on the GLMM with treatment group, treatment period and pre-dose baseline prodromal symptom intensity as categorical fixed effects. An unstructured covariance matrix was selected for the covariance matrix of the residual effects for the repeated measurements, corresponding to the two qualifying prodrome events, within a participant. Covariance structure of compound symmetry was used when the model did not converge. Similar analyses were performed by censoring data collected after rescue medication. All symptom efficacy analyses were done using SAS software, v.9·4 or newer (SAS Institute, Inc.).

### Standard protocol approvals, registration and patient consents

The Independent Ethics Committee or Institutional Review Board (IRB; that is, Advarra or University of Utah IRB) at each study site approved the study protocol, informed consent forms and recruitment materials before patient enrollment. The studies were conducted in accordance with the ICH guidelines, applicable regulations and the Declaration of Helsinki. All patients provided written informed consent before screening. The study is registered with ClinicalTrials.gov (NCT04492020). The study protocol and statistical analysis plan have been published^1^.

### Reporting summary

Further information on research design is available in the Nature Portfolio Reporting Summary linked to this article.

## Online content

Any methods, additional references, Nature Portfolio reporting summaries, source data, extended data, supplementary information, acknowledgements, peer review information; details of author contributions and competing interests; and statements of data and code availability are available at 10.1038/s41591-025-03679-7.

## Supplementary information

Supplementary Information Supplementary Tables 1–5 and Figs. 1 and 2.

Reporting Summary

## Data Availability

AbbVie is committed to responsible data sharing regarding the clinical trials that we sponsor. This includes access to anonymized, individual and trial-level data (analysis datasets), as well as other information (for example, protocols, clinical study reports or analysis plans), as long as the trials are not part of an ongoing or planned regulatory submission. This includes requests for clinical trial data for unlicensed products and indications. These clinical trial data can be requested by any qualified researchers who engage in rigorous, independent, scientific research and will be provided after review and approval of a research proposal, Statistical Analysis Plan and execution of a Data Sharing Agreement. Data requests can be submitted at any time after approval in the USA and Europe and after acceptance of this manuscript for publication. The data will be accessible for 12 months, with possible extensions considered. For more information on the process or to submit a request, visit the link https://vivli.org/ourmember/abbvie, then select ‘Home’.

## References

[1] GBD 2019 Diseases Injuries Collaborators. Global burden of 369 diseases and injuries in 204 countries and territories, 1990-2019: a systematic analysis for the Global Burden of Disease Study 2019. *Lancet* **396**, 1204–1222 (2020). 33069326 10.1016/S0140-6736(20)30925-9 PMC7567026

[2] Goadsby, P. J. et al. Pathophysiology of migraine: a disorder of sensory processing. *Physiol. Rev.* **97**, 553–622 (2017). 28179394 10.1152/physrev.00034.2015 PMC5539409

[3] Ferrari, M. D. et al. Migraine. *Nat. Prim.* **8**, 2 (2022). 10.1038/s41572-021-00328-4 35027572

[4] Headache Classification Committee of the International Headache Society (IHS). The International Classification of Headache Disorders, 3rd edition. *Cephalalgia* **38**, 1–211 (2018). 10.1177/0333102417738202 29368949

[5] Ashina, M. Migraine. *N. Engl. J. Med.* **383**, 1866–1876 (2020). 33211930 10.1056/NEJMra1915327

[6] Goadsby, P. J., Edvinsson, L. & Ekman, R. Vasoactive peptide release in the extracerebral circulation of humans during migraine headache. *Ann. Neurol.* **28**, 183–187 (1990). 1699472 10.1002/ana.410280213

[7] Goadsby, P. J. & Edvinsson, L. The trigeminovascular system and migraine: studies characterizing cerebrovascular and neuropeptide changes seen in humans and cats. *Ann. Neurol.* **33**, 48–56 (1993). 8388188 10.1002/ana.410330109

[8] Karsan, N., Bose, P. & Goadsby, P. J. The migraine premonitory phase. *Continuum (Minneap. Minn.)* **24**, 996–1008 (2018). 30074545 10.1212/CON.0000000000000624

[9] Giffin, N. J. et al. Premonitory symptoms in migraine: an electronic diary study. *Neurology* **60**, 935–940 (2003). 12654956 10.1212/01.wnl.0000052998.58526.a9

[10] Kelman, L. The premonitory symptoms (prodrome): a tertiary care study of 893 migraineurs. *Headache* **44**, 865–872 (2004). 15447695 10.1111/j.1526-4610.2004.04168.x

[11] Schoonman, G. G., Evers, D. J., Terwindt, G. M., van Dijk, J. G. & Ferrari, M. D. The prevalence of premonitory symptoms in migraine: a questionnaire study in 461 patients. *Cephalalgia* **26**, 1209–1213 (2006). 16961788 10.1111/j.1468-2982.2006.01195.x

[12] Cuvellier, J. C., Mars, A. & Vallee, L. The prevalence of premonitory symptoms in paediatric migraine: a questionnaire study in 103 children and adolescents. *Cephalalgia* **29**, 1197–1201 (2009). 19811504 10.1111/j.1468-2982.2009.01854.x

[13] Karsan, N., Prabakhar, P. & Goadsby, P. J. Characterising the premonitory stage of migraine in children: a clinic-based study of 100 patients in a specialist headache service. *J. Headache Pain* **14**, 17 (2016). 10.1186/s10194-016-0689-7 PMC5074936 27770403

[14] Maniyar, F. H., Sprenger, T., Monteith, T., Schankin, C. & Goadsby, P. J. Brain activations in the premonitory phase of nitroglycerin triggered migraine attacks. *Brain* **137**, 232–242 (2014). 24277718 10.1093/brain/awt320

[15] Schulte, L. H., Mehnert, J. & May, A. Longitudinal neuroimaging over 30 days: temporal characteristics of migraine. *Ann. Neurol.* **87**, 646–651 (2020). 32031707 10.1002/ana.25697

[16] Messina, R., Rocca, M. A., Goadsby, P. J. & Filippi, M. Insights into migraine attacks from neuroimaging. *Lancet Neurol.* **22**, 834–846 (2023). 37478888 10.1016/S1474-4422(23)00152-7

[17] Burstein, R., Blake, P., Schain, A. & Perry, C. Extracranial origin of headache. *Curr. Opin. Neurol.* **30**, 263–271 (2017). 28248698 10.1097/WCO.0000000000000437 PMC6051727

[18] Waelkens, J. Domperidone in the prevention of complete classical migraine. *Br. Med. J.* **284**, 944 (1982). 6802362 10.1136/bmj.284.6320.944 PMC1496523

[19] Massiou, H. Dihydroergotamine nasal spray in prevention and treatment of migraine attacks: two controlled trials versus placebo. *Cephalalgia* **7**, 440–441 (1987).

[20] Dodick, D. W. et al. Ubrogepant for the treatment of migraine attacks during the prodrome: a phase 3, multicentre, randomised, double-blind, placebo-controlled, crossover trial in the USA. *Lancet* **402**, P2307–P2316 (2023). 10.1016/S0140-6736(23)01683-5 37979595

[21] Moore, E. et al. Characterization of ubrogepant: a potent and selective antagonist of the human calcitonin gene-related peptide receptor. *J. Pharmacol. Exp. Ther.* **373**, 160–166 (2020). 10.1124/jpet.119.261065 31992609

[22] Goadsby, P. J. et al. Efficacy of ubrogepant for the treatment of migraine symptoms during the prodrome (premonitory phase): results from the PRODROME trial. *Neurology* **100**, S47.002 (2023).

[23] Lipton, R. B. et al. Improvement in patient-reported outcomes when ubrogepant is administered during the migraine prodrome (premonitory phase): results from the PRODROME trial. *Headache*10.1212/WNL.0000000000202049 (2023).

[24] Gowers, W. R. *A Manual of Diseases of the Nervous System* (P. Blakiston, Son & Co, 1899).

[25] Blau, J. N. Migraine prodromes separated from the aura: complete migraine. *Br. Med. J.* **21**, 658–660 (1980). 10.1136/bmj.281.6241.658 PMC1714112 7437756

[26] Drummond, P. D. & Lance, J. W. Neurovascular disturbances in headache patients. *Clin. Exp. Neurol.* **20**, 93–99 (1984). 6568949

[27] Quintela, E., Castillo, J., Munoz, P. & Pascual, J. Premonitory and resolution symptoms in migraine: a prospective study in 100 unselected patients. *Cephalalgia* **26**, 1051–1060 (2006). 16919055 10.1111/j.1468-2982.2006.01157.x

[28] Laurell, K. et al. Premonitory symptoms in migraine: a cross-sectional study in 2714 persons. *Cephalalgia* **36**, 951–959 (2016). 26643378 10.1177/0333102415620251

[29] Gago-Veiga, A. B. et al. To what extent are patients with migraine able to predict attacks? *J. Pain Res.* **11**, 2083–2094 (2018). 30310310 10.2147/JPR.S175602 PMC6166762

[30] Liveing, E. On megrim, sick-headache, and some allied disorders, a contribution to the pathology of nerve-storms. *Ind. Med. Gaz.* **8**, 305–306 (1873).

[31] Karsan, N. & Goadsby, P. J. Biological insights from the premonitory symptoms of migraine. *Nat. Rev. Neurol.* **14**, 699–710 (2018). 30448858 10.1038/s41582-018-0098-4

[32] Martins-Oliveira, M., Akerman, S., Tavares, I. & Goadsby, P. J. Neuropeptide Y inhibits the trigeminovascular pathway through NPY Y1 receptor: implications for migraine. *Pain* **157**, 1666–1673 (2016). 27023421 10.1097/j.pain.0000000000000571 PMC4949002

[33] Beck, B. Neuropeptide Y in normal eating and in genetic and dietary-induced obesity. *Philos. Trans. R. Soc. Lond. B Biol. Sci.* **361**, 1159–1185 (2006). 16874931 10.1098/rstb.2006.1855 PMC1642692

[34] Martins-Oliveira, M., Akerman, S., Holland, P. R., Tavares, I. & Goadsby, P. J. Pharmacological modulation of ventral tegmental area neurons elicits changes in trigeminovascular sensory processing and is accompanied by glycemic changes: Implications for migraine. *Cephalalgia* **42**, 1359–1374 (2022). 36259130 10.1177/03331024221110111 PMC9638709

[35] Schulte, L. H. & May, A. The migraine generator revisited: continuous scanning of the migraine cycle over 30 days and three spontaneous attacks. *Brain* **139**, 1987–1993 (2016). 27190019 10.1093/brain/aww097

[36] Maniyar, F. H., Sprenger, T. & Goadsby, P. J. Photic hypersensitivity in the premonitory phase of migraine—a positron emission tomography study. *Eur. J. Neurol.* **21**, 1178–1183 (2014). 24780052 10.1111/ene.12451

[37] Hitomi, S., Kross, K., Kurose, M., Porreca, F. & Meng, I. D. Activation of dura-sensitive trigeminal neurons and increased c-Fos protein induced by morphine withdrawal in the rostral ventromedial medulla. *Cephalalgia* **37**, 407–417 (2017). 27155000 10.1177/0333102416648655

[38] Marciszewski, K. K. et al. Changes in brainstem pain modulation circuitry function over the migraine cycle. *J. Neurosci.* **38**, 10479–10488 (2018). 30341182 10.1523/JNEUROSCI.1088-18.2018 PMC6596255

[39] Humphrey, P. P. A. et al. Serotonin and migraine. *Ann. N.Y. Acad. Sci.* **600**, 587–598 (1990). 2252337 10.1111/j.1749-6632.1990.tb16912.x

[40] Limmroth, V. et al. Changes in cerebral blood flow velocity after treatment with sumatriptan or placebo and implications for the pathophysiology of migraine. *J. Neurol. Sci.* **138**, 60–65 (1996). 8791240 10.1016/0022-510x(95)00344-2

[41] Hoskin, K. L., Kaube, H. & Goadsby, P. J. Sumatriptan can inhibit trigeminal afferents by an exclusively neural mechanism. *Brain* **119**, 1419–1428 (1996). 8931567 10.1093/brain/119.5.1419

[42] Charles, A. Vasodilation out of the picture as a cause of migraine headache. *Lancet Neurol.* **12**, 419–420 (2013). 23578774 10.1016/S1474-4422(13)70051-6

[43] Nicolodi, M. et al. Sumatriptan pharmacokinetics in the spinal fluid following oral administration of the drug. *Cephalalgia* **20**, 272 (2000).

[44] Rubio-Beltran, E., Labastida-Ramirez, A., Villalon, C. M. & MaassenVanDenBrink, A. Is selective 5-HT1F receptor agonism an entity apart from that of the triptans in antimigraine therapy? *Pharmacol. Ther.* **186**, 88–97 (2018). 29352859 10.1016/j.pharmthera.2018.01.005

[45] Goadsby, P. J. et al. Orally administered atogepant was efficacious, safe, and tolerable for the prevention of migraine: results from a phase 2b/3 study. *Neurology* **92**, S17.001 (2019).

[46] Clemow, D. B. et al. Lasmiditan mechanism of action—review of a selective 5-HT1F agonist. *J. Headache Pain* **21**, 71 (2020). 32522164 10.1186/s10194-020-01132-3 PMC7288483

[47] Hostetler, E. D. et al. In vivo quantification of calcitonin gene-related peptide receptor occupancy by telcagepant in rhesus monkey and human brain using the positron emission tomography tracer [^11^C]MK-4232. *J. Pharmacol. Exp. Ther.* **347**, 478–486 (2013). 23975906 10.1124/jpet.113.206458

[48] Ho, T. W. et al. Efficacy and tolerability of MK-0974 (telcagepant), a new oral antagonist of calcitonin gene-related peptide receptor, compared with zolmitriptan for acute migraine: a randomised, placebo-controlled, parallel-treatment trial. *Lancet* **372**, 2115–2123 (2008). 19036425 10.1016/S0140-6736(08)61626-8

[49] Giffin, N. J., Lipton, R. B., Silberstein, S. D., Olesen, J. & Goadsby, P. J. The migraine postdrome: an electronic diary study. *Neurology* **87**, 309–313 (2016). 27335112 10.1212/WNL.0000000000002789 PMC4955275

[50] International Conference on Harmonisation of Technical Requirements for Registration of Pharmaceuticals for Human Use. *Statistical Principles for Clinical Trials E9* (International Conference on Harmonisation, 1998).

