# Supplementary material for: Ubrogepant for the treatment of migraine prodromal symptoms: an exploratory analysis from the randomized phase 3 PRODROME trial
Source: Nat Med. 2025 May 12;31(7):2179–85. doi: 10.1038/s41591-025-03679-7 (PMC12283353; doi:10.1038/s41591-025-03679-7)

nature medicine

Supplementary information https://doi.org/10.1038/s41591-025-03679-7

Ubrogepant for the treatment of migraine prodromal symptoms: an exploratory analysis from the randomized phase 3 PRODROME trial

In the format provided by the authors and unedited

Supplementary Appendix

Table of Contents

Supplementary Tables

Supplementary Table 1. Summary of prodromal cognitive symptoms experienced at baseline in the double-blind period

Supplementary Table 2. Absence of most common prodromal symptoms of any intensity at timepoints post-dose by symptom prevalence at double-blind baseline[a]

Supplementary Table 3. Absence of most common prodromal symptoms of any intensity at timepoints post-dose by symptom prevalence at double-blind baseline,[a] censoring data collected after rescue medication

Supplementary Table 4. Rescue medication use (modified intent-to-treat population)

Supplementary Table 5. PRODROME Study Sites and Institutional Review Boards

Supplementary Figures

Supplementary Figure 1. Study design

Supplementary Figure 2. Prodromal symptoms of any intensity for the most common prodromal symptoms at post-dose timepoints censoring data collected after rescue medication

Supplementary Table 1. Summary of prodromal cognitive symptoms experienced at baseline in the double-blind period

Prodromal Symptom, *n* (%)[a]	Placebo (*n* = 449)	Ubrogepant 100 mg (*n* = 448)
Either difficulty concentrating or difficulty thinking, or both	148 (33.0)	144 (32.1)
Difficulty concentrating[b]	74 (16.5)	75 (16.7)
Mild	42 (56.8)	47 (62.7)
Moderate	29 (39.2)	24 (32.0)
Severe	3 (4.1)	4 (5.3)
Difficulty thinking[b]	47 (10.5)	41 (9.2)
Mild	25 (53.2)	24 (58.5)
Moderate	22 (46.8)	17 (41.5)
Severe	0	0
Both difficulty concentrating and difficulty thinking	27 (6.0)	28 (6.3)
Mild difficulty concentrating	13 (48.1)	15 (53.6)
Moderate difficulty concentrating	14 (51.9)	12 (42.9)
Severe difficulty concentrating	0	1 (3.6)
Mild difficulty thinking	15 (55.6)	16 (57.1)
Moderate difficulty thinking	11 (40.7)	11 (39.3)
Severe difficulty thinking	1 (3.7)	1 (3.6)

Prodromal symptoms identified at baseline are symptoms experienced by participants predose of reported qualifying prodrome events. Percentages of prodromal symptom incidence are based on overall treated events *n*, and percentages for the intensity levels are based on that symptom's *n*.
[a]Modified intent-to-treat population; the numbers and percentages represent participants with the prodromal symptom listed.
[b]Used for cognitive impairment efficacy analysis.

Supplementary Table 2. Absence of most common prodromal symptoms of any intensity at timepoints post-dose by symptom prevalence at double-blind baseline[a]

Prodromal Symptom/Baseline Prevalence	Pre-dose		Hour 1		Hour 2		Hour 3		Hour 4		Hour 6		Hour 8		Hour 24		Hour 48	
	PBO	Ubro	PBO	Ubro	PBO	Ubro	PBO	Ubro	PBO	Ubro	PBO	Ubro	PBO	Ubro	PBO	Ubro	PBO	Ubro
**Sensitivity to light, %**																		
N	273	273	258	252	257	256	257	250	249	250	240	242	238	239	244	248	221	220
Responder, *n*			12	12	32	50	60	81	88	112	107	140	120	170	187	203	184	194
Responder, %			4.7	4.8	12.5	19.5	23.3	32.4	35.3	44.8	44.6	57.9	50.4	71.1	76.6	81.9	83.3	88.2
OR (95% CI) vs PBO			1.08 (0.46, 2.51)		1.72 (1.13, 2.61)		1.58 (1.12, 2.23)		1.46 (1.10, 1.95)		1.75 (1.29, 2.38)		2.40 (1.75, 3.27)		1.37 (0.94, 1.99)		1.52 (1.00, 2.30)	
**Tired/sleepy/fatigue, %**																		
N	226	227	218	206	213	212	208	205	203	207	195	197	189	191	205	207	182	179
Responder, *n*			9	10	29	39	35	56	44	72	67	86	71	103	132	142	130	138
Responder, %			4.1	4.9	13.6	18.4	16.8	27.3	21.7	34.8	34.4	43.7	37.6	53.9	64.4	68.6	71.4	77.1
OR (95% CI) vs PBO			1.23 (0.47, 3.21)		1.43 (0.90, 2.25)		1.85 (1.17, 2.92)		1.93 (1.36, 2.75)		1.46 (1.06, 2.00)		1.87 (1.35, 2.57)		1.19 (0.85, 1.66)		1.22 (0.84, 1.79)	
**Neck pain/stiff neck, %**																		
N	180	180	170	168	168	169	170	159	169	163	162	152	159	149	162	168	153	144
Responder, *n*			5	7	19	26	27	46	37	63	57	73	66	75	103	120	118	112
Responder, %			2.9	4.2	11.3	15.4	15.9	28.9	21.9	38.7	35.2	48.0	41.5	50.3	63.6	71.4	77.1	77.8
OR (95% CI) vs PBO			1.43 (0.44, 4.60)		1.39 (0.75, 2.56)		2.04 (1.25, 3.32)		2.27 (1.51, 3.39)		1.68 (1.20, 2.35)		1.40 (0.95, 2.06)		1.42 (0.96, 2.12)		1.01 (0.65, 1.58)	
**Sensitivity to sound, %**																		
N	162	161	151	150	153	154	149	149	148	148	138	141	139	145	143	148	134	128
Responder, *n*			9	12	27	39	42	53	53	75	67	80	74	96	114	123	119	113
Responder, %			6.0	8.0	17.6	25.3	28.2	35.6	35.8	50.7	48.6	56.7	53.2	66.2	79.7	83.1	88.8	88.3
OR (95% CI) vs PBO			1.23 (0.59, 2.57)		1.59 (0.99, 2.55)		1.45 (0.98, 2.15)		1.97 (1.38, 2.80)		1.44 (0.99, 2.11)		1.75 (1.17, 2.62)		1.38 (0.88, 2.15)		0.93 (0.49, 1.77)	
**Dizziness/lightheaded/vertigo/imbalance, %**																		
N	139	130	129	120	127	123	130	122	131	122	124	119	124	111	130	122	118	105
Responder, *n*			14	12	33	36	38	48	56	61	65	74	80	80	107	108	99	94
Responder, %			10.9	10.0	26.0	29.3	29.2	39.3	42.7	50.0	52.4	62.2	64.5	72.1	82.3	88.5	83.9	89.5
OR (95% CI) vs PBO			0.96 (0.45, 2.02)		1.17 (0.72, 1.91)		1.60 (0.98, 2.60)		1.37 (0.87, 2.15)		1.45 (0.96, 2.19)		1.33 (0.82, 2.17)		1.82 (1.00, 3.30)		1.66 (0.85, 3.24)	
**Difficulty concentrating, %**																		
N	101	103	94	92	95	97	93	94	95	95	95	92	94	86	89	96	84	81
Responder, *n*			2	8	8	17	11	29	24	40	33	46	47	54	70	80	72	68
Responder, %			2.1	8.7	8.4	17.5	11.8	30.9	25.3	42.1	34.7	50.0	50.0	62.8	78.7	83.3	85.7	84.0
OR (95% CI) vs PBO			4.26 (1.17, 15.54)		2.54 (1.17, 5.51)		3.25 (1.52, 6.95)		2.14 (1.21, 3.78)		1.83 (1.05, 3.17)		1.55 (0.90, 2.64)		1.06 (0.63, 1.81)		0.75 (0.33, 1.74)	
**Difficulty thinking, %**																		
N	74	69	69	60	68	63	67	64	67	63	67	65	66	57	67	64	63	58
Responder, *n*			4	6	10	16	16	21	19	27	28	37	33	37	52	52	52	49
Responder, %			5.8	10.0	14.7	25.4	23.9	32.8	28.4	42.9	41.8	56.9	50.0	64.9	77.6	81.3	82.5	84.5
OR (95% CI) vs PBO			1.70 (0.42, 6.87)		1.83 (0.88, 3.79)		1.32 (0.75, 2.30)		2.04 (0.95, 4.39)		2.05 (1.14, 3.71)		1.92 (0.99, 3.69)		1.46 (0.82, 2.59)		0.96 (0.38, 2.39)	

Percentages were calculated as 100 × (*n*/*N*).

The ORs (95% CI) are based on a generalized linear mixed model with treatment group, treatment period, and pre-dose baseline prodromal symptom intensity as categorical fixed effects. An unstructured covariance matrix was selected for the covariance matrix of the residual effect for the repeated measurements (corresponding to the 2 qualifying prodrome events) within each participant. Covariance structure of compound symmetry was used when models did not converge.

[a]Top five individual symptoms and cognitive symptoms.

CI = confidence interval; *N* = number of participants with nonmissing prodromal symptom intensity assessment by timepoint, at predose *N* refers to numbers of participants with prodromal symptom; OR = odds ratio; PBO = placebo; Ubro = ubrogepant 100 mg.

Supplementary Table 3. Absence of most common prodromal symptoms of any intensity at timepoints post-dose by symptom prevalence at double-blind baseline,[a] censoring data collected after rescue medication

Prodromal Symptom/Baseline Prevalence	Pre-dose		Hour 1		Hour 2		Hour 3		Hour 4		Hour 6		Hour 8		Hour 24		Hour 48	
	PBO	Ubro	PBO	Ubro	PBO	Ubro	PBO	Ubro	PBO	Ubro	PBO	Ubro	PBO	Ubro	PBO	Ubro	PBO	Ubro
Sensitivity to light, %																		
N	273	273	255	245	235	237	219	224	193	220	177	206	164	202	154	188	131	169
Responder, *n*			12	12	31	46	52	72	72	102	80	123	86	147	116	159	107	149
Responder, %			4.7	4.9	13.2	19.4	23.7	32.1	37.3	46.4	45.2	59.7	52.4	72.8	75.3	84.6	81.7	88.2
OR (95% CI) vs PBO			1.10 (0.47, 2.56)		1.60 (1.04, 2.44)		1.48 (1.01, 2.17)		1.37 (0.99, 1.90)		1.76 (1.24, 2.50)		2.24 (1.57, 3.21)		1.72 (1.07, 2.78)		1.90 (1.14, 3.17)	
Tired/sleepy/fatigue, %																		
N	226	227	216	200	194	196	174	182	159	183	141	169	127	158	126	160	107	140
Responder, *n*			9	9	27	35	30	46	35	60	49	73	52	87	81	110	74	107
Responder, %			4.2	4.5	13.9	17.9	17.2	25.3	22.0	32.8	34.8	43.2	40.9	55.1	64.3	68.8	69.2	76.4
OR (95% CI) vs PBO			1.12 (0.43, 2.92)		1.36 (0.85, 2.16)		1.65 (0.98, 2.76)		1.66 (1.10, 2.48)		1.33 (0.92, 1.94)		1.68 (1.16, 2.43)		1.18 (0.76, 1.85)		1.42 (0.91, 2.21)	
Neck pain/stiff neck, %																		
N	180	180	169	164	152	162	142	148	126	149	116	131	102	127	87	126	82	105
Responder, *n*			5	7	19	25	23	42	30	58	40	64	49	65	58	92	64	84
Responder, %			3.0	4.3	12.5	15.4	16.2	28.4	23.8	38.9	34.5	48.9	48.0	51.2	66.7	73.0	78.0	80.0
OR (95% CI) vs PBO			1.47 (0.45, 4.75)		1.24 (0.67, 2.30)		1.97 (1.16, 3.35)		2.04 (1.28, 3.23)		1.72 (1.16, 2.57)		1.08 (0.68, 1.72)		1.29 (0.74, 2.24)		1.16 (0.61, 2.20)	
Sensitivity to sound, %																		
N	162	161	148	144	133	138	121	128	108	126	90	118	87	118	80	109	72	96
Responder, *n*			9	11	24	36	34	48	41	64	44	68	49	79	62	89	62	84
Responder, %			6.1	7.6	18.0	26.1	28.1	37.5	38.0	50.8	48.9	57.6	56.3	66.9	77.5	81.7	86.1	87.5
OR (95% CI) vs PBO			1.17 (0.54, 2.50)		1.62 (0.97, 2.70)		1.43 (0.92, 2.22)		1.59 (1.08, 2.34)		1.31 (0.82, 2.10)		1.32 (0.83, 2.10)		1.26 (0.69, 2.29)		1.07 (0.48, 2.38)	
Dizziness/lightheaded/vertigo/imbalance, %																		
N	139	130	127	117	116	112	110	107	108	107	95	101	90	94	82	94	69	83
Responder, *n*			13	12	32	33	35	43	46	54	51	64	59	69	66	82	56	73
Responder, %			10.2	10.3	27.6	29.5	31.8	40.2	42.6	50.5	53.7	63.4	65.6	73.4	80.5	87.2	81.2	88.0
OR (95% CI) vs PBO			1.02 (0.48, 2.19)		1.08 (0.64, 1.81)		1.46 (0.88, 2.43)		1.39 (0.86, 2.25)		1.34 (0.82, 2.19)		1.40 (0.77, 2.56)		1.80 (0.83, 3.89)		1.72 (0.75, 3.95)	
Difficulty concentrating, %																		
N	101	103	94	91	86	91	79	85	73	84	69	77	62	69	54	75	48	61
Responder, *n*			2	8	8	16	11	27	21	37	21	42	32	43	40	61	40	52
Responder, %			2.1	8.8	9.3	17.6	13.9	31.8	28.8	44.0	30.4	54.5	51.6	62.3	74.1	81.3	83.3	85.2
OR (95% CI) vs PBO			4.31 (1.18, 15.80)		2.26 (1.03, 4.98)		2.57 (1.25, 5.27)		1.69 (0.93, 3.07)		2.53 (1.29, 4.93)		1.17 (0.65, 2.10)		1.18 (0.66, 2.11)		0.96 (0.33, 2.80)	
Difficulty thinking, %																		
N	74	69	69	60	64	55	56	52	53	50	48	48	44	45	40	46	34	42
Responder, *n*			4	6	10	16	16	19	12	23	17	27	22	29	30	36	27	34
Responder, %			5.8	10.0	15.6	29.1	28.6	36.5	22.6	46.0	35.4	56.3	50.0	64.4	75.0	78.3	79.4	81.0
OR (95% CI) vs PBO			1.70 (0.42, 6.87		2.08 (0.99, 4.39)		1.18 (0.66, 2.12)		3.01 (1.37, 6.61)		2.54 (1.19, 5.42)		1.66 (0.82, 3.37)		1.60 (0.88, 2.92)		0.93 (0.28, 3.05)	

Percentages were calculated as 100 × (*n*/*N*).

The ORs (95% CI) are based on a generalized linear mixed model with treatment group, treatment period, and pre-dose baseline prodromal symptom intensity as categorical fixed effects. An unstructured covariance matrix was selected for the covariance matrix of the residual effect for the repeated measurements (corresponding to the 2 qualifying prodrome events) within each participant. Covariance structure of compound symmetry was used when models did not converge.

[a]Top five individual symptoms and cognitive symptoms.

CI = confidence interval; *N* = number of participants with nonmissing prodromal symptom intensity assessment by timepoint, at predose *N* refers to numbers of participants with prodrome symptom; OR = odds ratio; PBO = placebo; Ubro = ubrogepant 100 mg.

Supplementary Table 4. Rescue medication use (modified intent-to-treat population)

	Placebo (N = 449)	Ubrogepant 100 mg (N = 448)
Used rescue medication within 24 hours post dose, n (%)	177 (39.4)	97 (21.7)
Odds ratio (95 CI%)		0.42 (0.32, 0.55)
Used rescue medication within 48 hours post dose, n (%)	186 (41.4)	103 (23.0)
Odds ratio (95 CI%)		0.42 (0.32, 0.54)

Rescue medication referred to the participants' own medications used for the acute treatment of migraine headache. In the double-blind treatment period, participants were allowed to use rescue medication for headaches that developed following a qualifying prodrome event, but were not to use rescue medication for mild headaches in the first 24 hours postdose.

Supplementary Table 5. PRODROME study sites and institutional review boards

Study Site	Study Site Location	IRB	IRB Location
Minneapolis Clinic of Neurology	Golden Valley, Minnesota	Advarra IRB	Columbia, Maryland
Research Trials Worldwide	Humble, Texas	Advarra IRB	Columbia, Maryland
Colorado Springs Neurological Associates	Colorado Springs, Colorado	Advarra IRB	Columbia, Maryland
University of Cincinnati Department of Psychiatry and Behavioral Neuroscience	Cincinnati, Ohio	Advarra IRB	Columbia, Maryland
Aventiv Research Inc	Columbus, Ohio	Advarra IRB	Columbia, Maryland
Suncoast Clinical Research, Inc	New Port Richey, Florida	Advarra IRB	Columbia, Maryland
Clinical Research Institute, Inc	Minneapolis, Minnesota	Advarra IRB	Columbia, Maryland
Sentara Neurology Specialists	Virginia Beach, Virginia	Advarra IRB	Columbia, Maryland
The Medical Research Network, LLC	New York, New York	Advarra IRB	Columbia, Maryland
ForCare Clinical Research	Tampa, Florida	Advarra IRB	Columbia, Maryland
Raleigh Neurology Associates, PA	Raleigh, North Carolina	Advarra IRB	Columbia, Maryland
Charlottesville Medical Research Center, LLC	Charlottesville, Virginia	Advarra IRB	Columbia, Maryland
OK Clinical Research	Edmond, Oklahoma	Advarra IRB	Columbia, Maryland
Summit Research Network Inc (Oregon)	Portland, Oregon	Advarra IRB	Columbia, Maryland
DiscoveResearch, Inc	Bryan, Texas	Advarra IRB	Columbia, Maryland
Rochester Clinical Research, Inc	Rochester, New York	Advarra IRB	Columbia, Maryland
FutureSearch Trials of Dallas	Dallas, Texas	Advarra IRB	Columbia, Maryland
Clinical Research Atlanta	Stockbridge, Georgia	Advarra IRB	Columbia, Maryland
Health Research of Hampton Roads, Inc	Newport News, Virginia	Advarra IRB	Columbia, Maryland
Deaconess Clinic – Gateway	Newburgh, Indiana	Advarra IRB	Columbia, Maryland

Study Site	Study Site Location	IRB	IRB Location
Frontier Clinical Research, LLC	Smithfield, Pennsylvania	Advarra IRB	Columbia, Maryland
Lehigh Center for Clinical Research	Allentown, Pennsylvania	Advarra IRB	Columbia, Maryland
NeuroTrials Research Inc.	Atlanta, Georgia	Advarra IRB	Columbia, Maryland
Suburban Research Associates	Media, Pennsylvania	Advarra IRB	Columbia, Maryland
California Headache & Balance Center	Fresno, California	Advarra IRB	Columbia, Maryland
Meridian Clinical Research, LLC	Savannah, Georgia	Advarra IRB	Columbia, Maryland
Puget Sound Neurology	Tacoma, Washington	Advarra IRB	Columbia, Maryland
FutureSearch Trials of Neurology	Austin, Texas	Advarra IRB	Columbia, Maryland
iResearch Atlanta, LLC	Decatur, Georgia	Advarra IRB	Columbia, Maryland
Clinical Neuroscience Solutions, Inc	Jacksonville, Florida	Advarra IRB	Columbia, Maryland
USF Health Morsani Center for Advanced Healthcare	Tampa, Florida	Advarra IRB	Columbia, Maryland
Tidewater Integrated Medical Research	Virginia Beach, Virginia	Advarra IRB	Columbia, Maryland
Northwest Clinical Research Center	Bellevue, Washington	Advarra IRB	Columbia, Maryland
Abington Neurological Associates, Ltd	Abington, Pennsylvania	Advarra IRB	Columbia, Maryland
St. Joseph’s Hospital & Medical Center – Barrow Neurological Institute	Phoenix, Arizona	Advarra IRB	Columbia, Maryland
Seattle Clinical Research Center	Seattle, Washington	Advarra IRB	Columbia, Maryland
ClinPoint Trials	Waxahachie, Texas	Advarra IRB	Columbia, Maryland
Accel Research Sites – St Petersburg Clinical Research Unit	St Petersburg, Florida	Advarra IRB	Columbia, Maryland
Advanced Research Institute	Ogden, Utah	Advarra IRB	Columbia, Maryland
Thomas Jefferson University – Jefferson Headache Center	Philadelphia, Pennsylvania	Advarra IRB	Columbia, Maryland
CTI Clinical Research Center	Cincinnati, Ohio	Advarra IRB	Columbia, Maryland

Study Site	Study Site Location	IRB	IRB Location
Northwest Clinical Trials, Inc	Boise, Idaho	Advarra IRB	Columbia, Maryland
Clinical Neuroscience Solutions, Inc	Memphis, Tennessee	Advarra IRB	Columbia, Maryland
WR-ClinSearch, LLC	Chattanooga, Tennessee	Advarra IRB	Columbia, Maryland
Dent Neurosciences Research Center	Amherst, New York	Advarra IRB	Columbia, Maryland
Boston Clinical Trials	Boston, Massachusetts	Advarra IRB	Columbia, Maryland
Advanced Neurosciences Research, LLC	Fort Collins, Colorado	Advarra IRB	Columbia, Maryland
Clinical Neuroscience Solutions, Inc	Orlando, Florida	Advarra IRB	Columbia, Maryland
McFarland Clinic, PC	Ames, Iowa	Advarra IRB	Columbia, Maryland
Sun Valley Research Center, Inc	Imperial, California	Advarra IRB	Columbia, Maryland
WR-PRI, LLC	Newport Beach, California	Advarra IRB	Columbia, Maryland
Imaging & Neuroscience Center, Inc	Salt Lake City, Utah	University of Utah IRB	Salt Lake City, Utah
Bio Behavioral Health	Toms River, New Jersey	Advarra IRB	Columbia, Maryland
Kansas Institute of Research	Overland Park, Kansas	Advarra IRB	Columbia, Maryland
Princeton Center for Clinical Research	Skillman, New Jersey	Advarra IRB	Columbia, Maryland
Tekton Research, Inc.	Austin, Texas	Advarra IRB	Columbia, Maryland
Centex Studies	Houston, Texas	Advarra IRB	Columbia, Maryland
PMG Research of Charlotte, LLC	Charlotte, North Carolina	Advarra IRB	Columbia, Maryland
George J. Rederich M.D. Inc.	Torrance, California	Advarra IRB	Columbia, Maryland
KLR Business Group, Inc dba Arkansas Clinical Research	Little Rock, Arkansas	Advarra IRB	Columbia, Maryland
WR-PRI LLC	Los Alamitos, California	Advarra IRB	Columbia, Maryland
Michigan Headache & Neurological Institute	Ann Arbor, Michigan	Advarra IRB	Columbia, Maryland

Study Site	Study Site Location	IRB	IRB Location
StudyMetrix Research, LLC	Saint Peters, Missouri	Advarra IRB	Columbia, Maryland
Excell Research; Inc	Oceanside, California	Advarra IRB	Columbia, Maryland
Diablo Clinical Research, Inc	Walnut Creek, California	Advarra IRB	Columbia, Maryland
Clinical Trials of South Carolina	Charleston, South Carolina	Advarra IRB	Columbia, Maryland
Central New York Clinical Research	Manlius, New York	Advarra IRB	Columbia, Maryland
Health Awareness, Inc	Jupiter, Florida	Advarra IRB	Columbia, Maryland
Sensible Healthcare, LLC	Ocoee, Florida	Advarra IRB	Columbia, Maryland
Collective Medical Research	Prairie Village, Kansas	Advarra IRB	Columbia, Maryland
IPS Research Company	Oklahoma City, Oklahoma	Advarra IRB	Columbia, Maryland
Clinvest Research, LLC	Springfield, Missouri	Advarra IRB	Columbia, Maryland
Upstate Clinical Research Associates, LLC	Williamsville, New York	Advarra IRB	Columbia, Maryland
Birmingham Clinical Research Unit	Birmingham, Alabama	Advarra IRB	Columbia, Maryland
Premiere Research Institute at Palm Beach Neurology	West Palm Beach, Florida	Advarra IRB	Columbia, Maryland

Supplementary Figure 1. Study design.

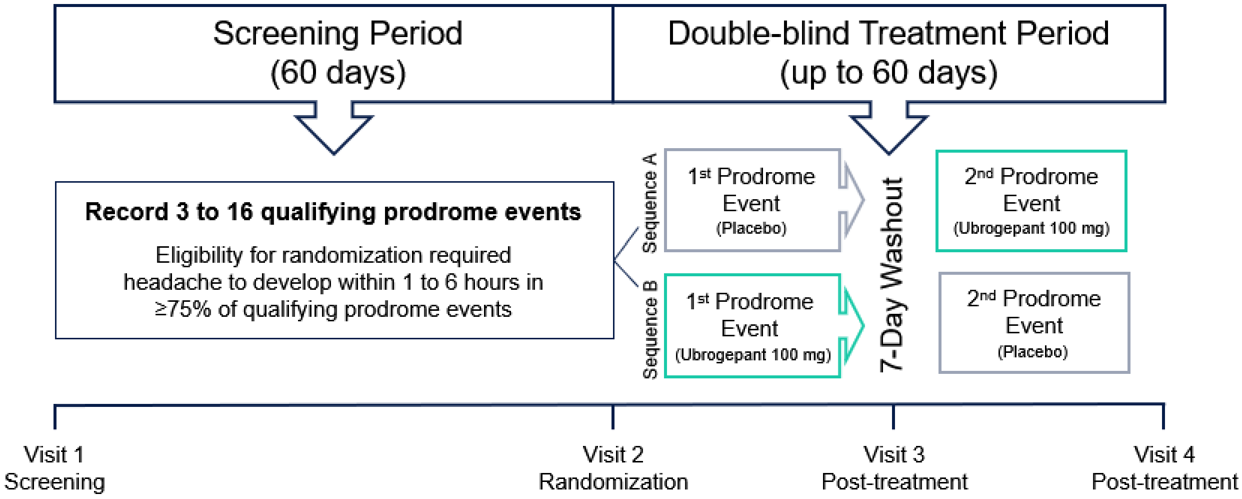

Supplementary Figure 2. Prodromal symptoms of any intensity for the most common prodromal symptoms at post-dose timepoints, censoring data collected after rescue medication. Shown are percent of participants with continuing prodromal symptoms post-dose censoring data collected after rescue medication for participants who reported the prodromal symptoms sensitivity to light (placebo, *n* = 273/449; ubrogepant 100 mg, *n* = 273/448) **(A)**, tired/sleepy/fatigue (placebo, *n* = 226/449; ubrogepant 100 mg, *n* = 227/448) **(B)**, neck pain/stiff neck (placebo, *n* = 180/449; ubrogepant 100 mg, *n* = 180/448) **(C)**, sensitivity to sound (placebo, *n* = 162/449; ubrogepant 100 mg, *n* = 161/448) **(D)**, dizziness/lightheaded/vertigo/imbalance (placebo, *n* = 139/449; ubrogepant 100 mg, *n* = 130/448) **(E)**, difficulty concentrating (placebo, *n* = 101/449; ubrogepant 100 mg, *n* = 103/448) **(F)**, and difficulty thinking (placebo, *n* = 74/449; ubrogepant 100 mg, *n* = 69/448) **(G)**.

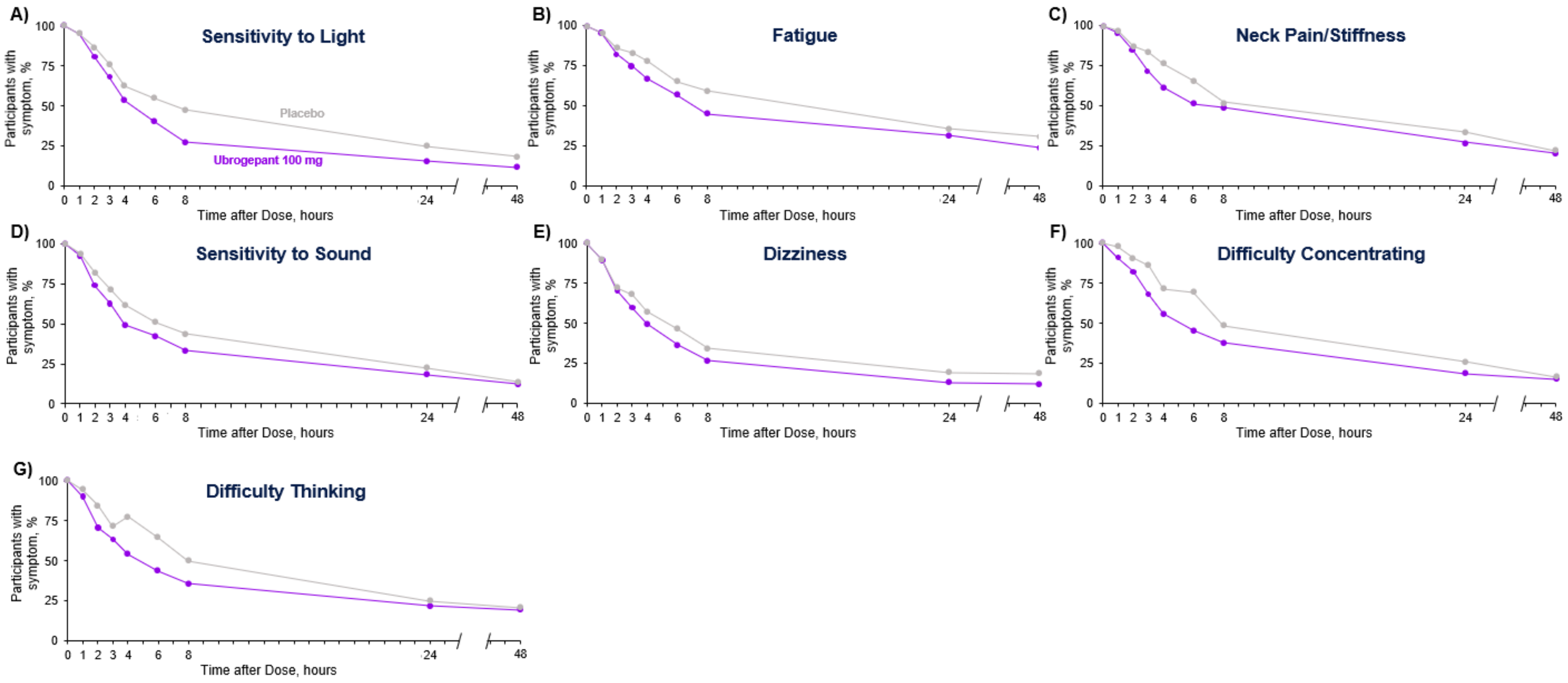

Supplement: Supplementary file 1 — Supplementary Tables 1–5 and Figs. 1 and 2. [file 41591_2025_3679_MOESM1_ESM.pdf]
