# Supplementary material for: Ubrogepant for the treatment of migraine prodromal symptoms: an exploratory analysis from the randomized phase 3 PRODROME trial
Source: Nat Med. 2025 May 12;31(7):2179–85. doi: 10.1038/s41591-025-03679-7 (PMC12283353; doi:10.1038/s41591-025-03679-7)
Supplement: Supplementary file 2 — Reporting Summary [file 41591_2025_3679_MOESM2_ESM.pdf]

nature portfolio

Corresponding author(s): Peter Goadsby

Last updated by author(s): Mar 25, 2025

Reporting Summary

Nature Portfolio wishes to improve the reproducibility of the work that we publish. This form provides structure for consistency and transparency in reporting. For further information on Nature Portfolio policies, see our Editorial Policies and the Editorial Policy Checklist.

Statistics

For all statistical analyses, confirm that the following items are present in the figure legend, table legend, main text, or Methods section.

n/a	Confirmed	
☐	☒	The exact sample size (*n*) for each experimental group/condition, given as a discrete number and unit of measurement
☐	☒	A statement on whether measurements were taken from distinct samples or whether the same sample was measured repeatedly
☐	☒	The statistical test(s) used AND whether they are one- or two-sided<br>*Only common tests should be described solely by name; describe more complex techniques in the Methods section.*
☐	☒	A description of all covariates tested
☒	☐	A description of any assumptions or corrections, such as tests of normality and adjustment for multiple comparisons
☐	☒	A full description of the statistical parameters including central tendency (e.g. means) or other basic estimates (e.g. regression coefficient) AND variation (e.g. standard deviation) or associated estimates of uncertainty (e.g. confidence intervals)
☒	☐	For null hypothesis testing, the test statistic (e.g. *F*, *t*, *r*) with confidence intervals, effect sizes, degrees of freedom and *P* value noted<br>*Give P values as exact values whenever suitable.*
☒	☐	For Bayesian analysis, information on the choice of priors and Markov chain Monte Carlo settings
☒	☐	For hierarchical and complex designs, identification of the appropriate level for tests and full reporting of outcomes
☒	☐	Estimates of effect sizes (e.g. Cohen's *d*, Pearson's *r*), indicating how they were calculated

Our web collection on statistics for biologists contains articles on many of the points above.

Software and code

Policy information about availability of computer code

Data collection	No software was used.
Data analysis	SAS software, version 9.4 or newer

For manuscripts utilizing custom algorithms or software that are central to the research but not yet described in published literature, software must be made available to editors and reviewers. We strongly encourage code deposition in a community repository (e.g. GitHub). See the Nature Portfolio guidelines for submitting code & software for further information.

Data

Policy information about availability of data

All manuscripts must include a data availability statement. This statement should provide the following information, where applicable:

- Accession codes, unique identifiers, or web links for publicly available datasets
- A description of any restrictions on data availability
- For clinical datasets or third party data, please ensure that the statement adheres to our policy

AbbVie is committed to responsible data sharing regarding the clinical trials we sponsor. This includes access to anonymized, individual, and trial-level data analysis data sets), as well as other information (eg, protocols, clinical study reports, or analysis plans), as long as the trials are not part of an ongoing or planned regulatory submission. This includes requests for clinical trial data for unlicensed products and indications. These clinical trial data can be requested by any qualified researchers who engage inrigorous, independent, scientific research, and will be provided following review and approval of a research proposal, Statistical Analysis

Plan (SAP), and execution of a Data Sharing Agreement (DSA). Data requests can be submitted at any time after approval in the US and Europe and after acceptance of this manuscript for publication. The data will be accessible for 12 months, with possible extensions considered. For more information on the process or to submit a request, visit the following link: https://vivli.org/ourmember/abbvie/ then select "Home".

Research involving human participants, their data, or biological material

Policy information about studies with human participants or human data. See also policy information about sex, gender (identity/presentation), and sexual orientation and race, ethnicity and racism.

Reporting on sex and gender	Reported in Table 1; sex was based on self-report
Reporting on race, ethnicity, or other socially relevant groupings	Reported in Table 1
Population characteristics	Described in Table 1
Recruitment	Recruitment materials were approved before patient enrollment (described in Methods section)
Ethics oversight	The Independent Ethics Committee or Institutional Review Board (ie, Advarra or University of Utah Institutional Review Board) at each study site approved the study protocol, informed consent forms, and recruitment materials before patient enrollment. The studies were conducted in accordance with the International Council for Harmonisation guidelines, applicable regulations, and the Declaration of Helsinki. All patients provided written informed consent before screening. The study is registered with ClinicalTrials.gov (NCT04492020). The study protocol and statistical analysis plan have been published.

Note that full information on the approval of the study protocol must also be provided in the manuscript.

Field-specific reporting

Please select the one below that is the best fit for your research. If you are not sure, read the appropriate sections before making your selection.

☒ Life sciences ☐ Behavioural & social sciences ☐ Ecological, evolutionary & environmental sciences

For a reference copy of the document with all sections, see nature.com/documents/nr-reporting-summary-flat.pdf

Life sciences study design

All studies must disclose on these points even when the disclosure is negative.

Sample size	modified intent-to-treat population, n=480; sample size determination was expanded in the statistical analysis section of the Methods
Data exclusions	Analyses were performed on observed data in participants who reported a prodromal symptom at baseline. Supplemental analyses excluding rescue medication censored data after reported rescue medication use in all participants.
Replication	The trial was not replicated
Randomization	Randomized
Blinding	Double-blind

Reporting for specific materials, systems and methods

We require information from authors about some types of materials, experimental systems and methods used in many studies. Here, indicate whether each material, system or method listed is relevant to your study. If you are not sure if a list item applies to your research, read the appropriate section before selecting a response.

Materials & experimental systems

n/a	Involved in the study
☒	☐ Antibodies
☒	☐ Eukaryotic cell lines
☒	☐ Palaeontology and archaeology
☒	☐ Animals and other organisms
☐	☒ Clinical data
☒	☐ Dual use research of concern
☒	☐ Plants

Methods

n/a	Involved in the study
☒	☐ ChIP-seq
☒	☐ Flow cytometry
☒	☐ MRI-based neuroimaging

Clinical data

Policy information about clinical studies

All manuscripts should comply with the ICMJE guidelines for publication of clinical research and a completed CONSORT checklist must be included with all submissions.

Clinical trial registration	NCT04492020
Study protocol	3110-302-002
Data collection	August 21, 2020 - April 19, 2022 in 75 research centers and headache clinics in the USA
Outcomes	The primary endpoint of the PRODROME trial was the absence of moderate or severe intensity headache within 24 hours post-dose. Prespecified additional endpoints were the absence of the five most common prodromal symptoms of any intensity and on cognitive impairment symptoms at each of the pre-specified timepoints (1, 2, 3, 4, 6, 8, 24 and 48 hours) regardless of the presence of headache. After identifying a "qualifying prodrome event" in which the participant was confident a headache would follow within 1 to 6 hours, the participant entered their premonitory (prodrome) symptoms in the eDiary and reported the presence or absence of those prodromal symptoms at the pre-specified time points.

Plants

Seed stocks	*Report on the source of all seed stocks or other plant material used. If applicable, state the seed stock centre and catalogue number. If plant specimens were collected from the field, describe the collection location, date and sampling procedures.*
Novel plant genotypes	*Describe the methods by which all novel plant genotypes were produced. This includes those generated by transgenic approaches, gene editing, chemical/radiation-based mutagenesis and hybridization. For transgenic lines, describe the transformation method, the number of independent lines analyzed and the generation upon which experiments were performed. For gene-edited lines, describe the editor used, the endogenous sequence targeted for editing, the targeting guide RNA sequence (if applicable) and how the editor was applied.*
Authentication	*Describe any authentication procedures for each seed stock used or novel genotype generated. Describe any experiments used to assess the effect of a mutation and, where applicable, how potential secondary effects (e.g. second site T-DNA insertions, mosiacism, off-target gene editing) were examined.*
